# Self-help materials for the prevention of smoking relapse: study protocol for a randomized controlled trial

**DOI:** 10.1186/1745-6215-13-69

**Published:** 2012-05-30

**Authors:** Fujian Song, Richard Holland, Garry R Barton, Max Bachmann, Annie Blyth, Viv Maskrey, Paul Aveyard, Stephen Sutton, Jo Leonardi-Bee, Thomas H Brandon

**Affiliations:** 1 Norwich Medical School, Faculty of Medicine and Health Science, University of East Anglia, Norwich NR4 7TJ, UK; 2 Primary Care Clinical Sciences, University of Birmingham, Birmingham, B15 2TT, UK; 3 Institute of Public Health, University of Cambridge, Cambridge, CB2 0SR, UK; 4 Division of Epidemiology & Public Health, University of Nottingham, Nottingham, NG5 1 PB, UK; 5 Department of Health Outcomes and Behavior, Moffitt Cancer Center, 4115 E. Fowler Ave., Tampa, FL, 33617, USA

**Keywords:** Smoking relapse, Self-help booklets, Effectiveness, Intervention

## Abstract

**Background:**

Most people who stop smoking successfully for a few weeks will return to smoking again in the medium term. There are few effective interventions to prevent this relapse and none used routinely in clinical practice. A previous exploratory meta-analysis suggested that self-help booklets may be effective but requires confirmation. This trial aims to evaluate the effectiveness and cost-effectiveness of a set of self-help educational materials to prevent smoking relapse in the National Health Service (NHS) Stop Smoking Service.

**Methods/design:**

This is an open, randomized controlled trial. The target population is carbon monoxide (CO) verified quitters at four weeks in the NHS stop smoking clinic (total sample size N = 1,400). The experimental intervention tested is a set of eight revised Forever Free booklets, including an introduction booklet and more extensive information on all important issues for relapse prevention. The control intervention is a leaflet that has no evidence to suggest it is effective but is currently given to some patients using NHS stop smoking services.

Two follow-up telephone interviews will be conducted at three and 12 months after the quit date. The primary outcome will be prolonged abstinence from months four to 12 with no more than five lapses, confirmed by a CO test at the 12-month assessment. The secondary outcomes will be seven-day self-report point prevalence abstinence at three months and seven-day biochemically confirmed point prevalence abstinence at 12 months. To assess cost-effectiveness, costs will be estimated from a health service perspective and the EQ-5D will be used to estimate the QALY (Quality Adjusted Life Year) gain associated with each intervention.

The comparison of smoking abstinence rates (and any other binary outcomes) between the two trial arms will be carried out using odds ratio as the outcome statistic and other related statistical tests. Exploratory subgroup analyses, including logistic regression analyses with interaction terms, will be conducted to investigate possible effect-modifying variables.

**Discussion:**

The possible effect of self-help educational materials for the prevention of smoking relapse has important public health implications.

**Trial registration:**

Current Controlled Trials ISRCTN36980856

## Background

Behavioral support and pharmacotherapy are effective to help smokers who are motivated to quit to do so [1-5]. However, relapse rates following these interventions are high [6]. According to data from English Stop Smoking Services, about 50% of smokers who set a quit date stopped smoking at four weeks [7], but 75% of the four-week quitters go back to regular smoking between four and 52 weeks [8]. The long-term success rates still make these interventions highly cost-effective but there is a need to find effective interventions to reduce relapse rates after the initial treatment episode. A Cochrane systematic review of trials of interventions for smoking relapse prevention [9,10] concluded that ‘there is insufficient evidence to support the use of any specific intervention for helping smokers who have successfully quit for a short time to avoid relapse’ [11]. Therefore, the current smoking cessation guidelines do not recommend any specific interventions for smoking relapse prevention [12,13]. A survey of smoking cessation professionals found that the uncertain evidence base about effectiveness was an important barrier to the use of relapse prevention interventions [14].

### Findings from systematic reviews

Psycho-educational interventions for smoking relapse prevention are complex healthcare interventions that usually contain several interacting components and involve changes of people’s behaviors [15,16]. The new Medical Research Council (MRC) guidance on development and evaluation of complex interventions in health stresses that ‘a good theoretical understanding is needed of how the intervention causes change’ [16]. Pawson *et al*. recommended a method of ‘realist review’, which emphasizes the explicit consideration of theories underlying complex interventions [17]. We conducted a theory-guided research synthesis of 49 trials on psycho-educational interventions for smoking relapse prevention in an updated systematic review [18,19]. Most interventions in the trials included were at least partly based on the cognitive-behavioral approach to coping skills training [20].

With coping skills training, participants are trained to identify situations with high risks of smoking relapse (such as going out with friends or feeling frustrated) and to develop and practice skills to cope with such situations. Therefore, the effectiveness of coping skills training for relapse prevention will depend on (1) the delivery and receipt of interventions, (2) the acquisition of coping skills by quitters and (3) the use of such skills in high risk situations. This delineation of the mechanisms whereby coping skills training interventions work suggests that the following mediating process is likely to be important: acquisition of skills to identify and cope with high-risk situations and the actual use of coping skills when required. However, only a few trials have reported on these processes [19]. For example, only two trials reported the actual use of coping skills by participants. They showed that those participants in skills training and abstainers may be more likely to have used their newly acquired coping skills than those who relapsed [21,22].

The effectiveness of coping skills training differs between population subgroups. Our meta-analysis found that coping skills training for smoking relapse prevention was ineffective for pregnant or postpartum quitters, hospitalized ex-smokers, forced short-term quitters, and smokers with mental health illnesses or drug abusers. For quitters from general communities, coping skills training interventions were not effective for current smokers who were motivated to quit (odds ratio (OR) = 0.96, 95% confidence interval (CI) 0.82 to 1.12) or for those who had quit smoking for less than one week (OR = 1.09, 95% CI 0.88 to 1.36). However, coping skills training interventions significantly reduced smoking relapse in community quitters who, at baseline, had been able to quit for at least one week (OR = 1.44, 95% CI 1.14 to 1.81) [19].

These findings show that the timing of relapse prevention is important and that coping skills training seems effective in secured quitters who are highly motivated to remain abstinent. Clearly, the acquisition of coping skills alone is not sufficient and only those who use these skills really benefit [23]. This evidence can be interpreted in terms of the theoretical mechanisms of coping skills training for relapse prevention.

Furthermore, interventions of using self-help materials seem as effective as interventions based on individual or group counseling. Five trials of coping skills training in community quitters who stopped smoking for at least one week were further separated into two subgroups: trials of self-help material and trials of counseling. The pooled OR of coping skills training is 1.46 (95% CI: 1.05 to 2.05) for self-help material trials and 1.41 (95% CI 1.02 to 1.94) for counseling trials [19]. A more recently published meta-analysis also found that written self-help materials were the only type of relapse prevention intervention for unaided quitters with established efficacy [24].

### Forever free booklets

Self-guided educational materials for skills training seem as effective as, but cheaper than, more expensive face-to-face counseling sessions and so seem likely to be more cost-effective. Brandon *et al*. have developed a series of eight booklets to be used as self-help materials for smoking relapse prevention [25,26]. Forever Free booklets for smoking relapse prevention have been evaluated by Brandon *et al*. in two randomized controlled trials in the United States [25,26] (included in the meta-analysis above). Volunteers who had quit smoking unaided were randomized to receive either all eight booklets or only the introductory booklet. Participants who received all eight booklets had a lower rate of smoking relapse than participants who received only a single booklet (the introduction booklet). The more recent trial suggested that repeated mailing (high contact) was no more effective than massed mailing (low contact) of the eight booklets [26]. Brandon *et al*. suggested that their two studies may have under-estimated the true effectiveness of Forever Free booklets, because participants in the control group received the introduction booklet that provided a summary of all relevant skills. The use of the Forever Free booklets for smoking relapse prevention was likely to be highly cost-effective (US $83 to $160 per QALY gained) [26].

### Objectives

Existing trials on coping skills training for smoking relapse prevention in community quitters were mostly conducted in the United States and recruited participants mainly by advertisement in newspapers. It is uncertain whether the results of our meta-analysis [19] and individual trials by Brandon *et al*. [25,26] are generalizable to four-week quitters who used the NHS stop smoking services. A more recently completed report of health technology assessment also recommended further research on the effectiveness of self-help interventions for smoking relapse prevention [27].

The current study aims to evaluate the effectiveness and cost-effectiveness of self-help materials (Forever Free booklets) for the prevention of smoking relapse in four-week quitters who have used NHS stop smoking services. By the end of the proposed trial, we will know whether short-term quitters who have used self-help material (Forever Free booklets) for relapse prevention have a lower rate of smoking relapse at 12 months, as compared with short-term quitters in the control group.

## Methods/design

**Figure 1:**
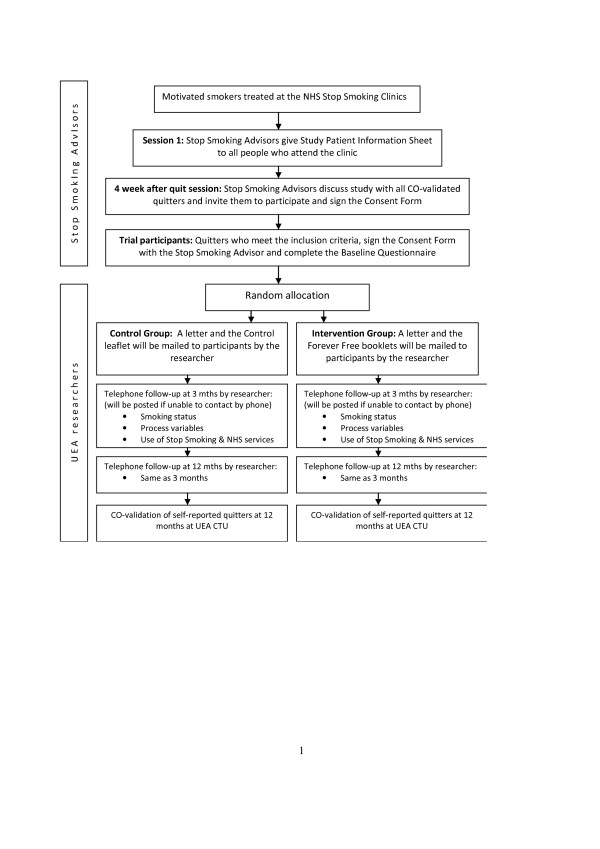
Flow Diagram – trial of self-help educational material (Forever Free booklets) for smoking relapse prevention.

This is an open, randomized controlled trial to evaluate the effectiveness of self-help educational materials (Forever Free booklets) for the prevention of smoking relapse, compared to a smoking cessation booklet used currently. Figure 1 shows the flow diagram of the trial.

This will be an open trial, without attempts to blind investigators and patients after randomization. Because the outcome assessor and trial participants know the allocated intervention, bias may be introduced into the results. However, evidence suggests that the risk of bias may be much reduced in trials with objectively assessed outcomes [28]. In this proposed trial, the primary outcome will be biochemically verified smoking abstinence at 12 months, which can be considered as an objectively assessed outcome.

### Setting

We will recruit four-week quitters in NHS Stop Smoking clinics (SmokeFree Norfolk, Norfolk Community Health & Care). In comparison with the rest of England, Norfolk has a relatively high proportion of people aged 65 and above (21.4% versus 16.5% in 2010), and a higher proportion of white people (94.3% versus 87.5% in 2009). Of those who set a quit date in 2010/2011, the percentage of successful quitters was 52% as compared with an average of 49% in England [29].

The investigated self-help educational materials will be sent to trial participants for their use at home. At the final follow-up (12 months from quit date), we will invite those who are still not smoking to have a breath CO test to confirm the self reported status of non-smoking. The test will be carried out by a researcher from University of East Anglia (UEA) in Norwich. People may come to a clinic at UEA or we can visit them at home for this test.

### Participant recruitment and randomization

Current smokers are referred to the NHS Stop Smoking Clinics from various sources (for example, general practitioners (GPs), self-referral, and so on). Clients who contact the Stop Smoking Service are given an appointment for assessment with a stop smoking advisor, either individually or in group sessions. Following assessment, the client typically receives weekly behavioral support, focused on withdrawal oriented therapy, with medication to reduce craving and withdrawal. The total contact time for each client is at least 1.5 hours from pre-quit preparation to four weeks after quitting. About 50% of clients who set a quit date are successful at four weeks but most (75%) of them relapse by 12 months [8]. Interventions for smoking relapse or maintenance of abstinence are not routinely provided, although stop smoking services will support renewed quit attempts for relapsed smokers.

The target population of the proposed trial is four-week quitters treated in the NHS Stop Smoking Service clinics. Stop smoking advisors will give a Patient Information Sheet to all expected quitters at week two or three following the quit date. At the final follow-up session (four weeks post-quit) in the NHS Stop Smoking Service clinic, the advisor will again explain the nature of the trial, to CO-verified quitters only, answer questions from them, and invite them to participate in the trial by signing the consent form. Clients who have failed to quit will not be included.

After eligible quitters have signed the consent form, stop smoking advisors will help them complete the baseline questionnaire and send it to the trial coordinator at UEA. The trial coordinator will randomly allocate participants to two groups (the control group and the relapse prevention group) and send them the corresponding self-help material by post. Quitters in the control group will receive a letter and the currently used leaflet, ‘Learning to Stay Stopped’. Quitters in the intervention group will receive a letter and all eight Forever Free booklets (see section Planned interventions for more details on these booklets).

The random allocation of participants into the two arms will be carried out by using a computerized allocation system provided by the Norwich Clinical Research and Trial Unit (CRTU). This allocation of trial participants is ‘concealed’ because the recruitment of quitters occurs before the random allocation.

It is necessary to avoid possible information contamination across the trial arms and ‘non-independence’ between members of the same family who live at the same address. We will include only one member from the same family in the analysis. Where couples of the same family are recruited at the same time, we will randomly select only one of them into the trial. If the members of the same family use the NHS Stop Smoking Service at different times during the trial recruitment period, we will include only the first family member. To carry out exploratory analysis, we will record information on whether there are other family members who are excluded for this reason.

We will prevent multiple entries of the same people who make repeat attempts to quit during the trial period. Before recruiting a new short-term quitter, stop smoking advisors will check whether the quitter has already been included in the trial. In addition, the trial coordinator will also check to make sure no multiple entries exist for the same quitter.

### Planned interventions

The experimental intervention tested in the proposed trial is the full pack of eight Forever Free booklets. Booklet one is a brief summary of all relevant issues, including an introduction of nicotine dependence, the stages of smoking cessation, situations that are high risk for relapse, ways of coping with urges to smoke, the abstinence violation effect, and ways to handle an initial slip [26]. The remaining seven booklets provide more extensive information on important issues for relapse prevention and are entitled *Smoking Urges; Smoking and Weight; What if You Have a Cigarette?; Your Health; Smoking, Stress, and Mood; Lifestyle Balance;* and *Life Without Cigarettes*[26]. The booklets can be understood by people with a reading level of fifth to sixth grade in the United States (expected reading level for children of ten to 12 years old).

The original Forever Free Booklets are prepared for users in the United States. We will revise and update the booklets in places where judged necessary or helpful, to make the material more suitable to British users and the UK NHS. We have obtained permission from the copyright holders (H. Lee Moffitt Cancer Center and Research Institute, Tampa, FL). Members of the trial steering committee, project team, and three lay representatives will review and comment on the revised booklets.

During their modification, we will ensure the acceptability/understanding of the booklets to as wide a range of users as possible. For the proposed trial, the revised booklets will be available in the English language only because of the considerable cost implications to translate the booklets into other languages. If proved effective, the booklets can be translated into other languages or different media formats (for example, DVD video) for people who have difficulty in English reading. For participant recruitment, stop smoking advisors will find out whether the client is able to read and understand English.

After randomization, we will send a letter and the full pack of eight revised Forever Free booklets to participants in the intervention group and a currently used leaflet (‘Learning to Stay Stopped’) to participants in the control group. The leaflet (with eight pages in total) used in the control contains comprehensive but brief information on issues related to smoking relapse and provides brief recommendations on how to cope with cravings and tempting triggers.

### Inclusion and exclusion criteria

The target population of the proposed trial is four-week quitters treated in the NHS Stop Smoking Service clinics. The biochemically verified four-week quitter is defined as a treated smoker who reports abstinence from day 14 post-quit date to the four-week follow-up point (or within 25 to 42 days of the quit date) and who blows an exhaled CO reading of less than 10 ppm [30]. The inclusion and exclusion criteria are summarized below.

Inclusion criteria:CO-verified quitters at four weeks in the NHS Stop Smoking Service clinic who sign the consent form to participate in the trial.

· CO-verified quitters at four weeks in the NHS Stop Smoking Service clinic who sign the consent form to participate in the trial.

Exclusion criteria:Pregnant quitters will be excluded. The process of relapse in pregnant women is very different than that in non-pregnant smokers. According to the available research evidence [19], smoking relapse prevention intervention by coping skills training is ineffective for women who have stopped smoking during pregnancy.We will exclude quitters who are not able to read the educational material in English, because the revised booklets are currently available in English language only.We will exclude quitters from families at the same address, as a family member has already been included in the trial.

· Pregnant quitters will be excluded. The process of relapse in pregnant women is very different than that in non-pregnant smokers. According to the available research evidence [19], smoking relapse prevention intervention by coping skills training is ineffective for women who have stopped smoking during pregnancy.

· We will exclude quitters who are not able to read the educational material in English, because the revised booklets are currently available in English language only.

· We will exclude quitters from families at the same address, as a family member has already been included in the trial.

### Sample size

The abstinence rate of four-week quitters at 12 months is estimated to be 25.0% in the control and 32.4% in the intervention group (based on an OR of 1.44). Assuming alpha = 0.05 and 1-beta = 0.8 and a dropout rate of 15%, about 1,400 patients will be required in total (700 in each arm) [31]. To simplify the project management, we will recruit participants mainly from the core Stop Smoking clinics in Norfolk.

### Baseline and follow up data collection

At the four-week session following the quit date, stop smoking advisors will gather baseline information from participants who have consented to be in the trial. The information collected includes participants’ demographic characteristics (for example, age, gender, and occupation), heaviness of smoking index, smoking and quitting history, and level of confidence to remain abstinent.

The consensus statement of the Society for Research on Nicotine and Tobacco (SRNT) and the Russell Standard both suggest that prolonged abstinence should be the primary outcome of a smoking cessation trial [32,33]. The normal form of prolonged abstinence allows a two week grace period in which lapses do not invalidate abstinence following quit day to assess the outcome of aid to cessation trials. However, a relapse prevention intervention might reasonably prevent lapses (occasional smoking) from becoming relapses (fulltime smoking and abandonment of the quit). Therefore, we propose a two-month grace period during which lapses to smoking do not count against achieving abstinence. As participants will be four weeks abstinent on enrollment, this equates to the primary outcome being prolonged abstinence from three months after quit day to 12 months follow up. Following the Russell Standard, the primary outcome will be prolonged abstinence from months four to 12 with no more than five lapses, confirmed by CO < 10 ppm at the 12-month assessment. The secondary outcomes will be seven-day self-report point prevalence abstinence at three months and seven-day biochemically confirmed point prevalence abstinence at 12 months.

To assess cost-effectiveness, costs will be estimated from a health service perspective. We will collect data on the resource use associated with self-help materials (including intellectual property, adaptation, printing and postage), any additional stop smoking services and cessation products at follow-up interviews. Our hypothesis is that the intervention, if effective, will improve abstinence rates, reduce repeated use of stop smoking services and might reduce use of other health care. Other resources which might be affected by the intervention will also be monitored, including GP visits and hospital admissions. The EQ-5D [34] will be used to estimate the benefits in terms of the QALY gain associated with each intervention during the study period.

At three months after quit day (two months after enrollment), a researcher from UEA will telephone participants primarily to assess process measures, that is, receipt, liking of, and use of the manuals (in the intervention group) and to assess key skills the manuals try to teach (in both the intervention and control groups). If the intervention is ineffective, this will help us to explain whether the intervention was used but people did not acquire the skills or whether applying the skills was ineffective in preventing relapse. This early follow-up contact is important, considering the high risk of relapse during the first few months.

The second and final follow up telephone call (conducted by a researcher from UEA) will take place at 12 months after quit day (11 months after enrollment), primarily to assess the primary and secondary outcomes, although we will once again test for skills acquired. Participants who meet the self-report criteria for at least seven-day abstinence will be invited to attend a local center to prove this by exhaled CO. People may come to a clinic at UEA or we can visit them at home for this test. We will offer a shopping voucher (£20) to each of the participants who have attended the CO-test at 12 months.

To minimize loss of follow-up, we will include the alternative contact telephone for each trial participant and ask the most appropriate time for the telephone contact. If necessary, we will make multiple attempts to contact trial participants. We will send the follow-up questionnaire by post with a freepost return envelope to those we are not able to contact by telephone.

### Data analysis plan

We will develop a trial database to maintain data from baseline and follow-up questionnaires. Data analyses will be conducted using STATA software. The comparison of smoking abstinence rates (and any other binary outcomes) between the two trial arms will be carried out using OR (and 95% confidence intervals) as the outcome statistic.

The primary outcome will be prolonged abstinence from months four to 12 with no more than five lapses, confirmed by CO < 10 ppm at the 12-month assessment. Participants who decline biochemical verification or who do not respond to follow up will be counted as smokers but those who have died or genuinely moved away will be disregarded from the numerator and denominator. If we detect differences in missing data we will assess the robustness of our primary analyses by using imputation methods such as pattern-mixture models [35].

Exploratory subgroup analyses, including logistic regression analyses with interaction terms, will be conducted to investigate possible effect-modifying variables, including age, gender, socio-economic status, level of nicotine dependence, number of prior quit attempts, and use of pharmacological interventions. These exploratory analyses have low statistical power and are also likely to yield false positive findings so the results will need to be interpreted with great caution.

The analysis of mediating variables will examine hypothesized mechanisms of the intervention. The intervention using the Forever Free booklets teaches skills based on the cognitive-behavioral approach. The effectiveness of coping skills training for relapse prevention depends on (1) the adequate delivery and receipt of the booklets, (2) the acquisition of coping skills by quitters and (3) the application of such skills in high-risk situations. This intervention mechanism suggests certain important process variables that should be investigated in the trial. At the follow-up interviews, we will at first ask trial participants whether they have received the booklets, whether they have read the booklets (and how much time spent and how many booklets they looked at). We will then investigate whether the use of booklets helped in the acquisition of coping skills by the participants, in terms of improved capability to identify risky situations and to know more appropriate ways of handling urges to smoke again. Thirdly, we will ask the trial participants whether they have actually applied the skills learned from the booklets. Finally, we will invite the trial participants to give an overall assessment of the usefulness of the booklets. We will summarize data collected in tables and plots to examine associations between smoking abstinence and important mediating variables (for example, level of the use of booklets, actual application of coping skills in high-risk situations).

We will also use more complex methods for the exploratory mediation analysis according to MacKinnon and Fairchild [36]. As an example, the Single-Mediator model consists of three equations:

(1) Y=i1+cX+e1;

(2) Y=i2+c'X+bM+e2;

(3) M=i3+aX+e3;

where Y is the dependent variable (smoking relapse), X is the independent variable (the intervention), and M is the mediating variable (for example, time spent on reading the booklets, acquisition of coping skills, and so on). Then, the mediating effect of M could be quantified by a*b (or c'-c). These exploratory mediation analyses will help understand how and why the intervention works or does not work.

Using data on time to the first event of smoking relapse, survival curves of the intervention and control arm will be compared by using Cox regression analysis as secondary analyses. This might reveal patterns indicating that relapse is postponed, which could suggest that ‘top up interventions’ might be helpful.

Using data from the trial, we will calculate the mean incremental cost for those in the intervention arm (Forever Free booklets) compared to the control arm. Similarly, the incremental effect of the intervention will be equivalent to the difference in the 12-month quit rate between the two arms. If dominance does not occur (that is, if one intervention is more costly and more effective) [37], the incremental cost and incremental effect will be used to calculate the cost required to have one more long-term quitter with the provision of the Forever Free booklets. The cost-effectiveness analysis will be based on individual level costs and outcome data, with uncertainty expressed as cost effectiveness acceptability curves and if appropriate, CIs for the incremental cost effectiveness ratio. As part of a cost-utility analysis, the incremental QALY gain associated with the intervention will be estimated, based on EQ-5D data collected in the trial and findings from existing studies of relevant economic evaluations [38-40].

### Ethical arrangements

No adverse effects or harm on the target population or society could be expected from the intervention. We will provide sufficient information for four-week quitters to consider whether they would like to participate in the trial and recruit only those who sign the consent form. Data on individual participants will remain strictly confidential. Only researchers directly involved in the trial will have access to participants' personal data during the study. Research ethical approval has been granted by East of England Research Ethics Committee (11/EE/0091).

### Project management

A Trial Steering Committee will be established to provide overall supervision for the study on behalf of the National Institute for Health Research - Health Technology Assessment (NIHR HTA) program. The trial will be overseen by a trial management group (TMG), based at UEA, including all co-investigators. The TMG will meet every three months to monitor and manage the trial and review an ongoing CONSORT statement (including data on recruitment, intervention and follow-up). The Trial Project Team, consisting of the trial coordinator/RA, project secretary, and chief investigator, will meet at UEA weekly to monitor the day-to-day progress and management of the trial.

No adverse effects are expected to be associated with the educational booklets for smoking relapse prevention. This trial poses no substantial risks to participants and there would be no cause to propose stopping rules for premature closure of the trials. In addition, this trial is open, un-blinded. After a discussion with the NIHR HTA program, we decided not to have a separate Data Monitoring Committee (DMC). Data monitoring will be carried out by study team members (TMG) and be synchronized with Trial Steering Committee meetings.

### Project timetable and milestones

The proposed trial will last 36 months, including the revising and printing of relapse prevention booklets, patient recruitment, delivery of interventions, follow-up data collection, data analysis, and writing up report and papers. The proposed trial start date is 1 June 2011. We will need two months to revise and print the Forever Free booklets and to arrange trainings for stop smoking advisors who will be involved in the recruitment of four-week quitters. The recruitment of trial participants will start in August 2011 and last for 21 months until April 2013. This arrangement will cover the period in which quit attempts are most frequent (January to March) in both 2012 and 2013.

## Discussion

### Important public health implications

If proven effective, the use of the self-help booklets for smoking relapse prevention may result in more than 7,000 additional long-term quitters each year in England, which could be associated with 3,500 life-years saved [19]. In addition, the self-help educational material for smoking relapse prevention may be a cost-saving intervention by reducing repeat use of the NHS Stop Smoking Service and by reducing use of healthcare services for smoking-related illness. The prevention of smoking relapse in four-week quitters may contribute to a reduction in adult smoking rates from 21% in 2008 to 10% or less by 2020, a target set out in the new Tobacco Control Strategy for England [41].

### Major limitations of the study

Given the nature of interventions and methods for following up, it is impossible to confidently blind trial participants and outcome assessors. However, the primary outcome (CO-verified smoking abstinence at 12 months) is objectively measured, which may be less vulnerable than subjectively measured outcomes to bias due to lack of blinding [28].

We will conduct exploratory analyses to investigate factors associated with the effectiveness of self-help material for the prevention of smoking relapse. The study will investigate whether the use of the booklets helps the acquisition of coping skills and whether the skills learned have been applied when required. However, questions asked at the follow-up interviews at three and 12 months may not be sufficient for the purpose of process evaluations. Further qualitative research is required to provide more details on patient experience of the use of self-help educational materials for the prevention of smoking relapse.

### Trial status

Participant recruitment started in August 2011 and is expected to end in April 2013.

## Abbreviations

CI: confidence interval; CO: carbon monoxide; CRTU: Clinical Research and Trial Unit; DMC: Data Monitoring Committee; GP: general practitioner; MRC: Medical Research Council; NHS: National Health Service; OR: odds ratio; QALY: quality adjusted life years; SRNT: the Society for Research on Nicotine and Tobacco; TMG: trial management group; UEA: University of East Anglia.

## Competing interests

The authors declare that they have no competing interests.

## Authors’ contributions

FS, RH, GRB, MB, PA, SS, JLB, and THB contributed to the development of the trial protocol. AB and VM refined the participant recruitment process, revised the trial information sheet, consent forms and interview questionnaires. FS prepared the manuscript. All authors commented upon and approved the final manuscript.
